# Impact of Timing Surgical Fixation on Complications and Functional Outcomes in Ankle Fractures

**DOI:** 10.7759/cureus.75313

**Published:** 2024-12-08

**Authors:** Abdullah Bin Sahl, Ella Barrett, Salim M Abduljawad, Mohamed Komber, Muntadhir Al-Uzri, Rana Balvinder, Anand Pillai

**Affiliations:** 1 Trauma and Orthopaedics, Wythenshawe Hospital, Manchester University NHS Foundation Trust, Manchester, GBR; 2 Trauma and Orthopaedics, Royal College of Surgeons in Ireland, Dublin, IRL; 3 Trauma and Orthopaedics, University of Manchester, Manchester, GBR; 4 Trauma and Orthopaedics, Royal Preston Hospital, Lancashire Teaching Hospitals NHS Foundation Trust, Preston, GBR

**Keywords:** ankle and foot, ankle fracture, boast, guideline, manchester-oxford foot questionnaire, orthopaedic, the national institute for health and care excellence (nice), timing, trauma, waiting time

## Abstract

Introduction: This study aimed to assess whether adherence to the British Orthopaedic Association Standards for Trauma (BOAST) and National Institute for Health and Care Excellence (NICE) guidelines for ankle fractures is associated with reduced complication rates and improved functional outcomes.

Methods: A retrospective analysis was conducted of all patients who underwent surgical fixation for ankle fractures in August 2023 to January 2024 from an acute hospital. Statistical analyses were performed using IBM SPSS Statistics software, version 29 (IBM Corp., Armonk, NY), to explore the relationship between BOAST and NICE guideline adherence and the study outcomes.

Results: The analysis revealed that only 10% of patients underwent ankle surgical fixation in accordance with the BOAST and NICE guidelines, with the mean time for surgery from injury as 10.7 days for the remaining cohort. Mental health effects were also recognised, with 26.7% due to waiting times. The association between BOAST and NICE compliance and complication incidence was found to be non-significant (p = 0.414). Mean Manchester-Oxford Foot Questionnaire (MOXFQ) scores were lower in the BOAST and NICE-compliant groups. The correlation coefficient between surgical waiting time and MOXFQ scores was r = 0.007, demonstrating no significant correlation between the time to surgery from injury and functional outcomes.

Conclusions: This study highlights the challenges in adhering to BOAST and NICE guidelines for timely surgical intervention in ankle fractures, with only a small proportion of patients receiving surgery within the recommended timeframe. Larger-scale studies are required to substantiate the hypothesis that early surgical intervention, as per the guidelines, leads to improved outcomes and whether achievement of the guidelines is feasible or requires re-examination.

## Introduction

Ankle fractures are increasingly prevalent, particularly among elderly females and young males in England [1]. These fractures not only represent one of the most common types of fractures but also rank second in terms of hospital admissions compared to other fracture types [2]. In cases requiring surgical management, there is ongoing debate regarding the impact of delays in fixation on postoperative complications, such as infection, wound breakdown, and blister formation. While some argue that prolonged waiting times exacerbate these complications, others suggest that the delay may not significantly influence outcomes [3].

The British Orthopaedic Association Standards for Trauma (BOAST) guidelines for the management of ankle fractures were introduced in August 2016 [4] with the aim of reducing morbidity through the standardisation of treatment protocols [5]. This study focuses specifically on point 10 of the guideline, which recommends early fixation, preferably on the day of injury or the following day, in the majority of patients under 60 years of age with an unstable ankle mortise [4]. The BOAST guidelines obtain this recommendation from the National Institute for Health and Care Excellence (NICE), from the fractures (non-complex) assessment and management guideline, point 1.4.2, published in February 2016 [6]. Multiple factors contribute to delays and cancellations in surgery, including the prioritisation of more urgent cases, such as neck of femur fractures, trauma cases, and post-injury ankle swelling. Consequently, adhering to the guidelines’ recommendation for early fixation is not always feasible. The primary objective of this study was to assess whether adherence to this recommendation influences complication rates, functional recovery, and patient outcomes. Another overarching goal was to evaluate whether the guidelines should be revised to extend the recommended timeframe for surgical fixation in order to align with current pressures in orthopaedic surgical services while still ensuring optimal patient outcomes.

In addition, the study considered the confounding factors of diabetes, alcohol consumption, and smoking status. These factors are known to impair healing and increase the risk of complications in ankle fractures [7-9].

The study also explored the psychological impact of extended waiting times for surgery, particularly in terms of patients' mental health and their perception of whether delayed surgery prolonged their recovery process.

## Materials and methods

This study was conducted on patients who required surgical fixation for a closed ankle fracture between August 31st, 2023, and January 31st, 2024. Data were collected at the orthopaedic department of a single acute hospital retrospectively, and patients were approached via telephone for additional retrospective data collection. The study included patients of all ages, including those aged 60 and above, where conservative management with a cast was deemed unsuitable. Exclusion criteria comprised open ankle fractures, pilon fractures, cases requiring a hindfoot nail (as this affected ankle range of motion), and patients who could not be contacted. 

To identify eligible patients, daily trauma handover sheets from the specified period were reviewed, and suitable candidates were identified. These patients were then individually searched in the hospital database, and data were collected on the following variables: age, gender, date of injury, date of hospital presentation, date of surgery, type of surgery, outcomes, complications, need for additional surgery, comorbidities, smoking status, alcohol intake, management at presentation, and reasons for any surgical delays. The collected data was systematically entered into a spreadsheet.

Approval for using the Manchester-Oxford Foot Questionnaire (MOXFQ) in this study was obtained through a license from Oxford University. Thirty of the identified patients met the inclusion criteria, were successfully contacted, and consented to participate in the study. Each participant completed the MOXFQ, with the questionnaire's page one and page two (Appendix A). The raw scores were subsequently converted into metric scores ranging from 0 to 100. Zero indicates no pain or issues with the ankle, and 100 indicates severe pain that impacts all daily activities [10].

In addition to the MOXFQ, patients were asked supplementary questions concerning the impact of surgical waiting times on their mental health, the effect of recovery duration on mental health, and whether they believed that the waiting time for surgery had prolonged their recovery process.

The data were analysed using IBM SPSS Statistics software, version 29 (IBM Corp., Armonk, NY). Statistical analyses were performed to compare patients who adhered to the BOAST and NICE guidelines with those who did not. Data were also analysed in aggregates. Those who adhered to the guidelines had surgery on the day of injury or the following day. Descriptive statistics, including frequencies, means, medians, standard deviations, and ranges, were calculated for relevant variables. A chi-squared test was conducted to assess associations between adherence to the BOAST and NICE guidelines and complications, while a t-test was employed to evaluate differences in the MOXFQ scores between the adherence and non-adherence groups. A p-value of <0.05 was considered indicative of statistical significance, and confidence intervals were calculated.

## Results

Patient demographics

The key demographic characteristics of the 30 patients included in this study are summarised in Table 1.

**Table 1:** The baseline characteristics and demographics for this study cohort The layout of Table 1 is adapted from [5]. All data are presented in the form of the number of patients (and percentage of patients in parentheses), N (%), except for age, which is presented as mean age (and the standard deviation in parentheses). BOAST: British Orthopaedic Association Standards for Trauma; NICE: National Institute for Health and Care Excellence

		BOAST and NICE compliant: Mean age/N (SD/%)	BOAST and NICE non-compliant: Mean age/N (SD/%)	All patients: Mean age/N (SD/%)
Age (years)	Mean, Mean age (SD)	45.33 (14.572)	48.00 (21.674)	47.73 (20.892)
Sex	Female, N (%)	2 (66.7)	16 (59.3)	18 (60.0)
	Male, N (%)	1 (33.3)	11 (40.7)	12 (40.0)
Diabetes mellitus	Type 1 diabetic, N (%)	0 (0)	1 (3.70)	1 (3.33)
	Type 2 diabetic, N (%)	0 (0)	5 (18.5)	5 (16.7)
	Non-diabetic, N (%)	3 (100)	21 (77.8)	24 (80.0)
Alcohol intake	Over recommended limits, N (%)	1 (33.3)	11 (40.7)	12 (40.0)
	Within recommended limits, N (%)	2 (66.7)	16 (59.3)	18 (60.0)
Smoking status	Smoker, N (%)	1 (33.3)	6 (22.2)	7 (23.3)
	Ex-smoker, N (%)	0 (0)	5 (18.5)	5 (16.7)
	Non-smoker, N (%)	2 (66.7)	16 (59.3)	18 (60.0)

Surgical delays

**Figure 1:**
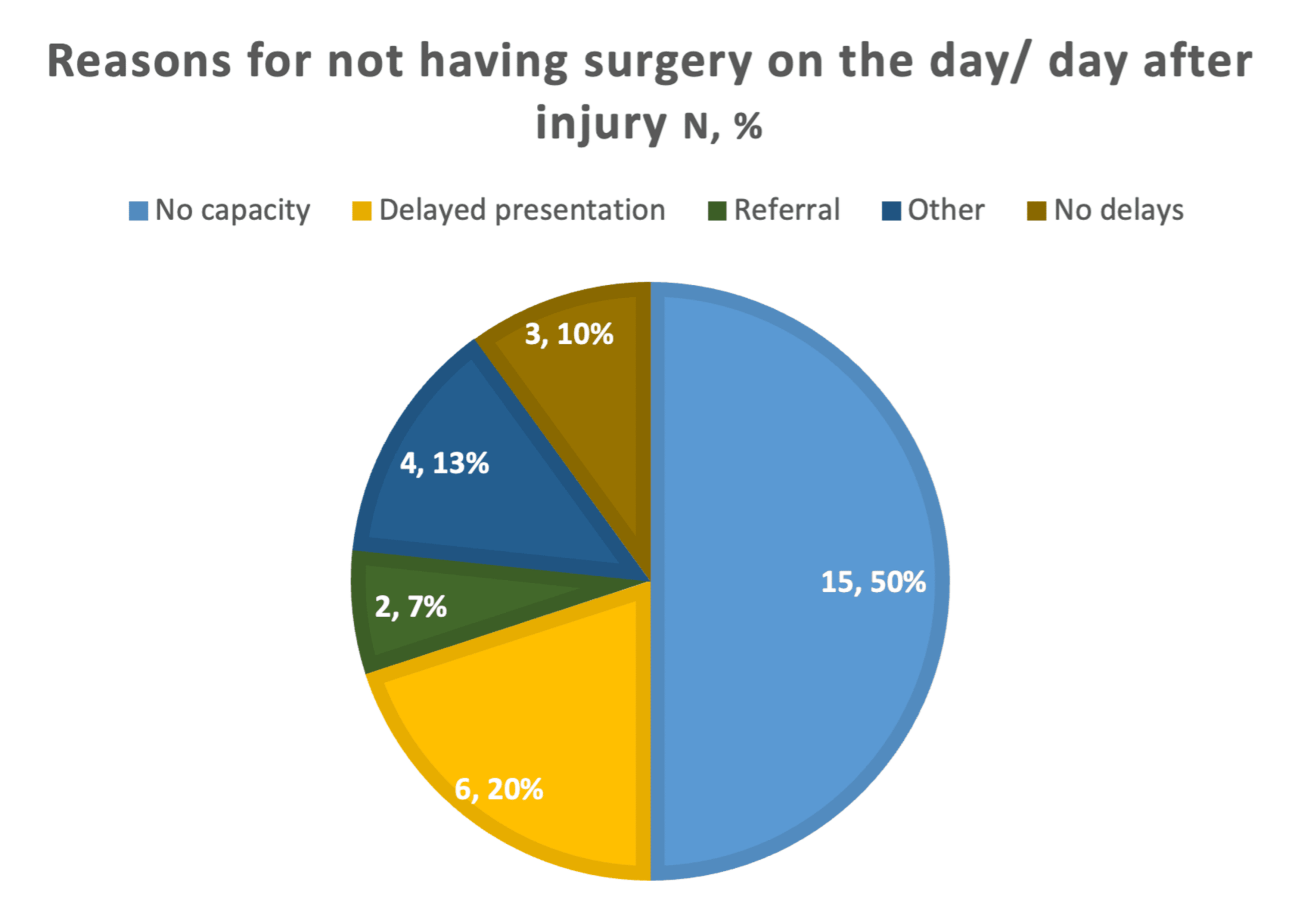
Pie chart showing the reasons why there was a delay to surgery and therefore why the BOAST and NICE guidelines (early fixation on the day or day after injury) were not followed. Values in the pie chart are presented as N, %. BOAST: British Orthopaedic Association Standards for Trauma; NICE: National Institute for Health and Care Excellence

Only three out of the 30 patients (10%) adhered to the BOAST and NICE guidelines by undergoing surgery on the day of injury or the following day. For the remaining 27 patients (90%) who did not meet these guidelines, the mean time from injury to surgery was 10.7 days, with a maximum delay of 22 days and a minimum delay of two days. Figure 1 outlines the reasons for non-compliance with the BOAST and NICE guidelines. The primary cause of surgical delay was a lack of theatre capacity, which accounted for 15 (50%) of the cases. This was due to various factors, including fully booked theatre lists, prioritisation of more urgent cases, and overrun surgeries. Six patients (20%) did not present to the hospital on the day of injury, often because they initially believed the injury to be minor or were intoxicated. Two patients (6.67%) were referred from another hospital, while four patients (13.3%) fell into the 'other' category, which included cases such as failed conservative management attempts and initial misdiagnosis of the fracture as an ankle sprain.

Swelling was also a contributing factor to surgical delays, though it was not the primary reason for the lack of early fixation. Swelling did prolong surgical waiting times; however, it was observed that significant swelling generally occurred 48 hours or more post injury. Thus, if surgery had been performed within the BOAST and NICE guidelines' timeframe, swelling would not likely have been an issue preoperatively. Although only a small number of patients underwent surgery in line with the guidelines, all patients who presented to this hospital received early treatment in the accident and emergency (A&E) department. This involved either reduction of the ankle under sedation with the application of a backslab or, alternatively, the provision of a walking boot with crutches and instructions to avoid weight-bearing.

Complications

**Figure 2:**
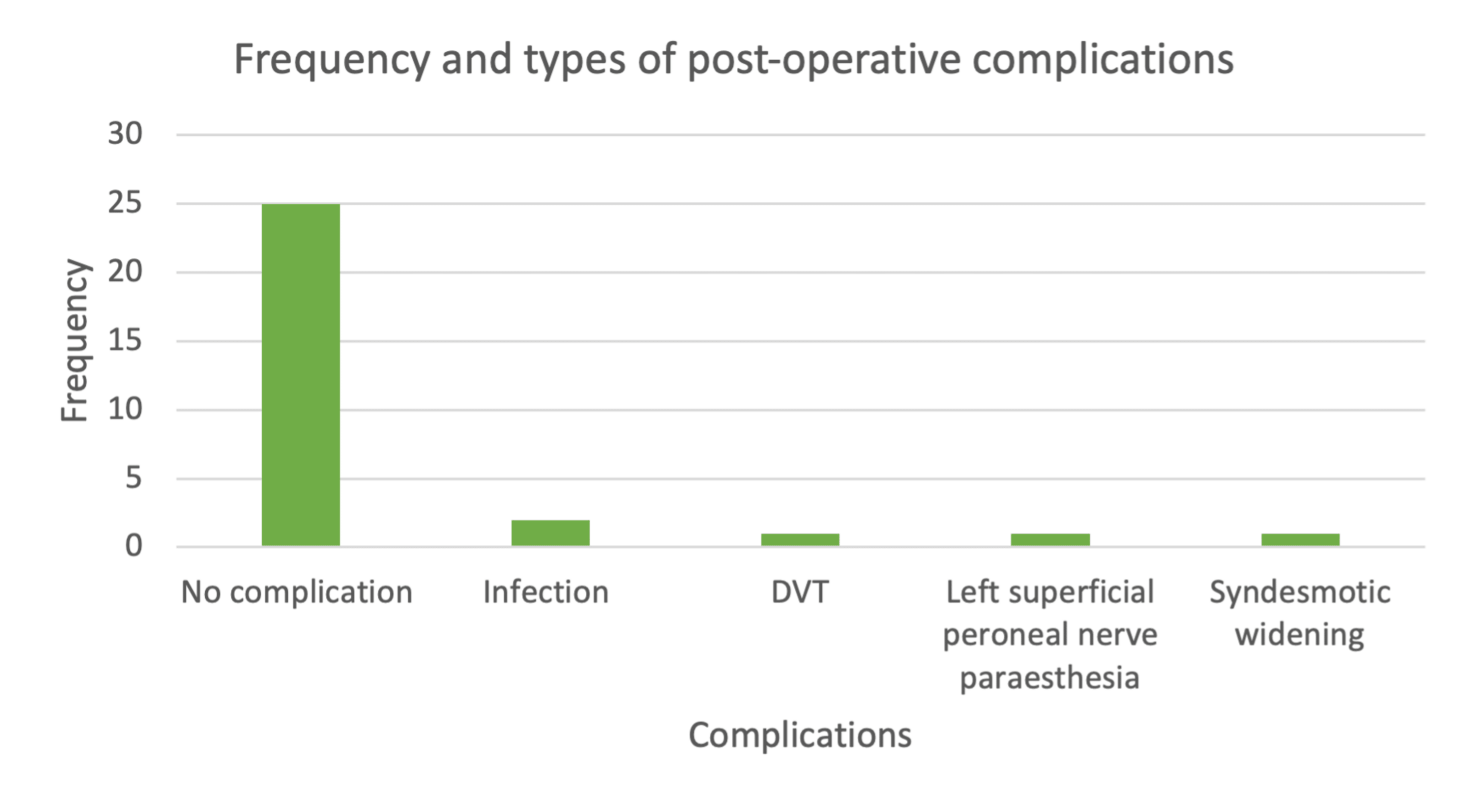
Bar chart demonstrating the frequency and types of complications that occurred in patients post-operatively DVT: deep vein thrombosis

All patients in this study underwent open reduction and internal fixation (ORIF). Post-operative complications were observed in five patients (16.7%), as detailed in Figure 2. The complications included infections in two patients (6.67%), deep vein thrombosis (DVT) in one patient (3.33%), superficial peroneal nerve paraesthesia in one patient (3.33%), and syndesmotic widening in one patient (3.33%). Three patients (10%) required additional surgery due to these complications. Notably, three out of the five patients with complications had undergone surgery within two to three days of injury. None of the patients who adhered to the BOAST and NICE guidelines experienced complications, whereas five (18.5%) of the non-compliant patients did. A chi-squared test was conducted to assess the association between BOAST and NICE compliance and the incidence of complications or infections with a significance level of p < 0.05, yielding p-values of 0.414 and 0.626, respectively. This indicates no statistical significance. However, due to the small sample size, these results lack sufficient power to draw definitive conclusions. Additional chi-squared tests were performed to evaluate the association between complications and individual risk factors such as diabetes, alcohol intake, and smoking status. The p-values for these analyses were 1.000, 0.317, and 0.966, respectively. This again showed no statistical significance (p < 0.05), but the small sample size limits the reliability of these findings.

The two patients who developed post-operative infections were further analysed for contributing risk factors. One patient had poorly controlled type 1 diabetes mellitus and diabetic neuropathy and was a smoker, all of which increased the risk of post-operative complications and infections. The second patient was an ex-smoker with no other significant risk factors, aside from not undergoing surgery within the guidelines' timeframe, although surgery was performed within 48 hours of injury.

The MOXFQ scores

**Figure 3:**
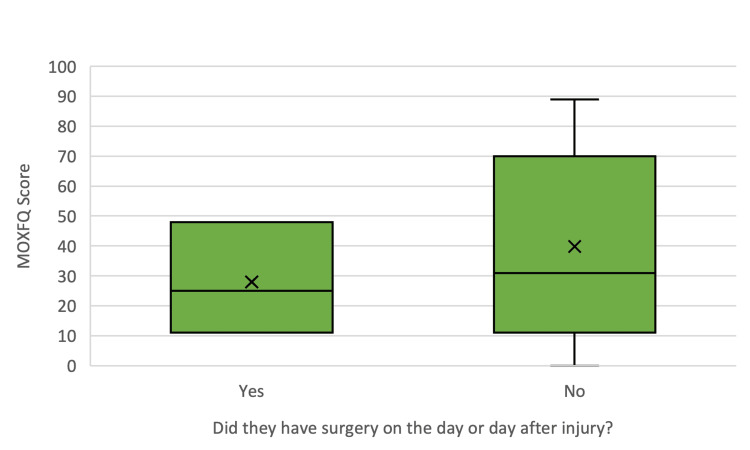
Box and whisker plot representing the data of the MOXFQ scores for the patients whose surgeries were BOAST and NICE compliant compared against those that were BOAST and NICE non-compliant. MOXFQ: Manchester-Oxford Foot Questionnaire; BOAST: British Orthopaedic Association Standards for Trauma; NICE: National Institute for Health and Care Excellence

The mean MOXFQ score for patients who complied with the BOAST and NICE guidelines was 28 ± 18.682, with confidence intervals for the mean ranging from -18.41 to 74.41. The median score was 25, with a minimum score of 11 and a maximum score of 48. In contrast, the mean MOXFQ score for patients who did not adhere to the BOAST and NICE guidelines was 39.81 ± 31.120, with confidence intervals of 27.5 to 52.13. The median score for this group was 31, with a minimum score of 0 and a maximum score of 89. This is illustrated in Figure 3. A two-sided t-test comparing these means resulted in a p-value of 0.528 (with significance levels as p < 0.05), indicating no statistical significance. However, the small sample size undermines the strength of these findings.

**Figure 4:**
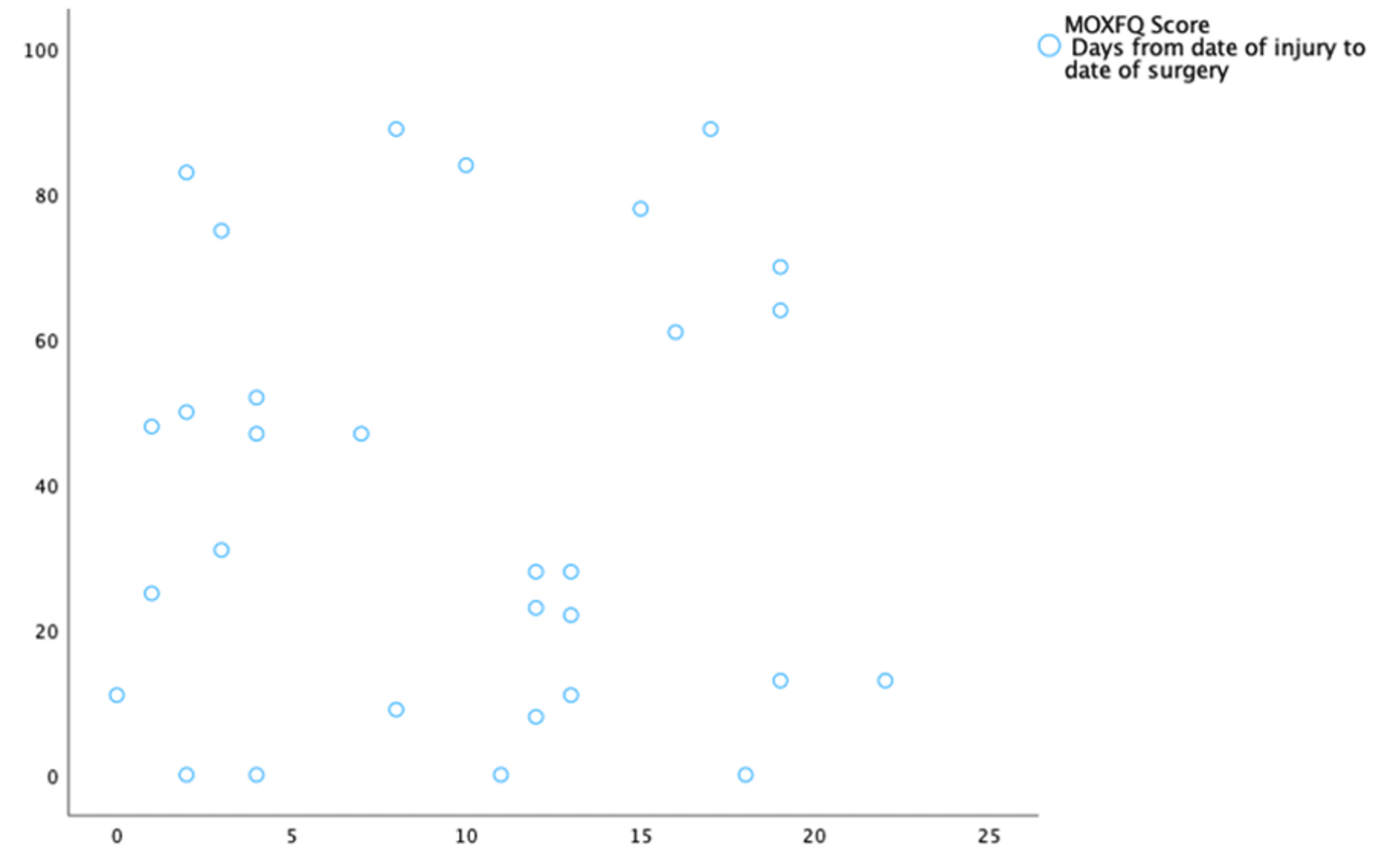
A simple scatterplot with the number of days from the date of injury to the date of surgery on the x-axis and the MOXFQ scores on the y-axis. It is used to illustrate whether there is a correlation between waiting times for surgery and functional recovery. MOXFQ: Manchester-Oxford Foot Questionnaire

As shown in Figure 3, the mean and median MOXFQ scores were lower for the BOAST and NICE-compliant group than for the non-compliant group. The range of scores in the non-compliant group was also broader. Notably, four out of the 27 non-compliant patients (14.8%) achieved a score of 0, indicating a return to full ankle function without pain. None of the compliant patients scored 0, with the lowest score being 11. The data for the interval between injury and surgery and the corresponding MOXFQ scores were plotted in a scatter graph to explore whether longer surgical waiting times negatively impacted functional recovery. This is presented in Figure 4. The estimated regression model for this analysis was y=38.320+0.32x. The coefficient of determination was 0, indicating that 0% of the variation in MOXFQ scores was explained by the waiting time for surgery. The correlation coefficient was r=0.007, further demonstrating no relationship between the time from injury to surgery and MOXFQ scores in this study.

Additionally, the data were analysed to assess whether patients who underwent surgery earlier had better outcomes than those who had surgery later. This was done by determining if the date of surgery correlated with MOXFQ scores. The correlation coefficient r=0.021 suggested no relationship between the date of surgery and MOXFQ scores. Once again, the small sample size limits the conclusiveness of these results.

Mental health and recovery

Eight patients (26.7%) reported that the waiting times for surgery negatively impacted their mental health. The primary reasons cited included the stress of being hospitalised or immobilised at home, the pain from the ankle fracture, and the frustration of surgery cancellations after being nil by mouth. The majority of patients (22, or 73.3%) reported no mental health impact from the surgical waiting times. However, 20 patients (66.7%) indicated that the recovery period affected their mental health due to the restrictions it imposed on their daily lives, while 10 patients (33.3%) did not report such an impact.

When asked whether the waiting time for surgery significantly affected their recovery, 26 patients (86.7%) responded negatively, three patients (10%) were unsure, and one patient (3.33%) believed it did. Most patients believed that their recovery was only prolonged by the number of days they waited for surgery, without a significant impact on overall progress. Some patients felt unqualified to assess whether the waiting times had affected their recovery. The one patient who believed that the delay had affected their recovery had initially undergone conservative management, leading them to think that immediate surgery would have resulted in a quicker recovery.

## Discussion

Principal findings

This study revealed that only 10% (N=3) of patients underwent surgery on the day of or the day following injury, in accordance with BOAST and NICE guidelines. The predominant reason for this delay affecting 50% (N=15) of patients was a lack of operating theatre capacity. Other contributing factors included delayed presentations and referrals. The limited size of the BOAST and NICE-compliant cohort constrained the ability to conduct robust statistical analyses and discern clear trends within the data. This finding highlights the current demands on orthopaedic surgical services and that existing infrastructure may not adequately support adherence to the guidelines. It is reasonable to infer that given sufficient theatre availability, a greater proportion of patients would receive surgery within the recommended timeframe. Importantly, all patients received some form of initial treatment upon presentation to the emergency department, such as ankle reduction, application of a back slab, or provision of a boot. This stabilises the ankle until surgery can be performed. Thus, extending the guideline for surgical fixation may be justifiable provided that initial stabilisation occurs within 24 hours of injury.

Complications occurred in 16.7% of patients (N=5 out of 30). None of these patients received surgery on the day of or the day after the injury, possibly indicating a higher complication rate among those not compliant with BOAST and NICE guidelines. Notably, three out of these five patients underwent surgery within two to three days of injury. While this could be considered early fixation, it is uncertain whether surgery performed 12-24 hours earlier would have prevented complications such as DVT and syndesmotic widening. Additionally, one patient experienced superficial peroneal nerve paraesthesia, likely attributable to the surgical procedure itself rather than the timing of the surgery. The infection rate was 6.67%, with two patients acquiring post-operative infections. Of these, one patient had multiple risk factors that likely contributed to the infection, making it improbable that surgical delay was the primary cause. The other patient underwent surgery within 48 hours of injury, suggesting that a minor delay relative to BOAST and NICE guidelines is unlikely to have influenced the outcome. Statistical analyses indicated no significant associations (p < 0.05) between complications (p=0.414) or infections (p=0.626) and compliance with the BOAST and NICE guidelines, although the small sample size limits the power of these findings. Overall, our results suggest that non-compliance with the BOAST and NICE guidelines for surgery on the day or the day after injury did not significantly impact post-operative complications.

Regarding functional recovery, the mean MOXFQ score was lower in the BOAST and NICE-compliant group compared to the non-compliant group. This implied better functional outcomes for those adhering to the guidelines. However, the t-test did not reveal a significant difference between the two means (p = 0.528, p < 0.05), and the small sample size diminishes the validity of this result. Interestingly, within the BOAST and NICE non-compliant groups, four patients achieved a MOXFQ score of 0. This indicates full functional recovery, compared to no patients achieving a score of 0 in the compliant group. The maximum MOXFQ score was also markedly higher in the non-compliant group (89 vs. 48), indicating a broader range of functional outcomes. This variation was anticipated due to the larger sample size of the non-compliant group. Correlational analysis between MOXFQ scores and the interval from injury to surgery revealed no significant relationship, nor was there an evident correlation between the timing of surgery and MOXFQ outcomes. This finding was unexpected, as patients with more extended recovery periods were presumed to have lower MOXFQ scores. The absence of clear trends in this data suggests that factors such as adherence to follow-up appointments, engagement in physiotherapy, and avoidance of further ankle trauma may play critical roles in functional recovery. Additionally, as the MOXFQ is a patient-reported outcome measure (PROM), variations in patient expectations and baseline mobility likely influenced the scores. Consequently, our data does not support the hypothesis that non-compliance with the BOAST and NICE guidelines significantly contributes to poorer functional outcomes. This conclusion aligns with previous studies by Conway et al. [11], who found no impact of surgical delay on mid-term outcomes, and by Pilskog et al. [12], who suggested that surgery within seven days post injury yields better outcomes. Considering these findings, it may be appropriate to extend the guideline for surgical fixation to within seven days of injury, potentially accommodating preoperative swelling reduction without compromising patient outcomes.

Mental health is a critical factor to consider when evaluating the potential extension of BOAST and NICE guidelines. Over a quarter of patients (N=8) reported that their mental health was negatively impacted by surgical delays, primarily due to pain and stress. While factors such as non-weight bearing, hospital admission, and surgical cancellations due to urgent cases are often unavoidable, improving pain management for patients awaiting surgery could mitigate preoperative distress. Additionally, two-thirds of patients (N=20) reported mental health challenges during recovery, citing limitations in daily activities, reduced social engagement, and inability to work as significant stressors. These challenges, while inherent to the recovery process following an ankle fracture, highlight the need for comprehensive post-operative support. Some patients also expressed self-consciousness about residual swelling, limping, or the need to alter footwear. This indicates that full functional recovery should consider the patient's ability to return to pre-injury activities, including wearing specific types of footwear.

Strengths and weaknesses

Given the increasing incidence of ankle fractures in England, auditing compliance with BOAST and NICE guidelines is essential. These guidelines are designed to optimise patient outcomes, and it is crucial that they are critically evaluated to determine their necessity or potential for modification. This study ensured data accuracy by manually reviewing each patient record without relying on coding and by cross-verifying information such as smoking status and diabetes diagnosis directly with patients. A comprehensive range of variables that could affect healing, functional recovery, and complication risk was considered. The use of the MOXFQ, a PROM, allowed for a more nuanced assessment of patient outcomes considering not just the range of motion but also pain, functional limitations, and their impact on daily life. However, the study's small sample size and the relatively short follow-up period (five to 10 months post surgery) limited the statistical power and prevented assessment of long-term functional outcomes, as some patients were still undergoing physiotherapy and showing improvement.

Areas for further research

To draw definitive conclusions regarding the impact of early fixation on complications and functional outcomes, this study should be replicated on a larger scale and over an extended timeframe. A prospective study design, possibly involving a research team to manage the substantial data collection required will be beneficial. Future research should also consider fracture type (e.g., trimalleolar vs. lateral malleolar) as a variable potentially influencing outcomes. The study should calculate both pre-operative and ≥12-month post-operative MOXFQ scores, allowing sufficient time for a full recovery and accurate final outcome assessment.

## Conclusions

Our study's finding that only 10% (N=3) of patients underwent surgery within the timeframe recommended by the BOAST and NICE guidelines highlights significant challenges in adhering to these standards due to the pressures in orthopaedic surgery. The observed higher rates of complications, infections, and suboptimal recovery in BOAST and NICE non-compliant patients suggest potential adverse outcomes associated with delayed surgery. However, after adjusting for other risk factors, no definitive evidence was found that surgical delay was the primary cause of these outcomes. We recommend conducting further studies with larger sample sizes and extended time frames. If future studies confirm that non-adherence does not significantly affect patient outcomes, it may be prudent to reconsider the current surgical timeframe, potentially extending it to within seven days post-injury to better accommodate practical surgical constraints while maintaining adherence to the guidelines.

## Figures and Tables

**Figure 5:**
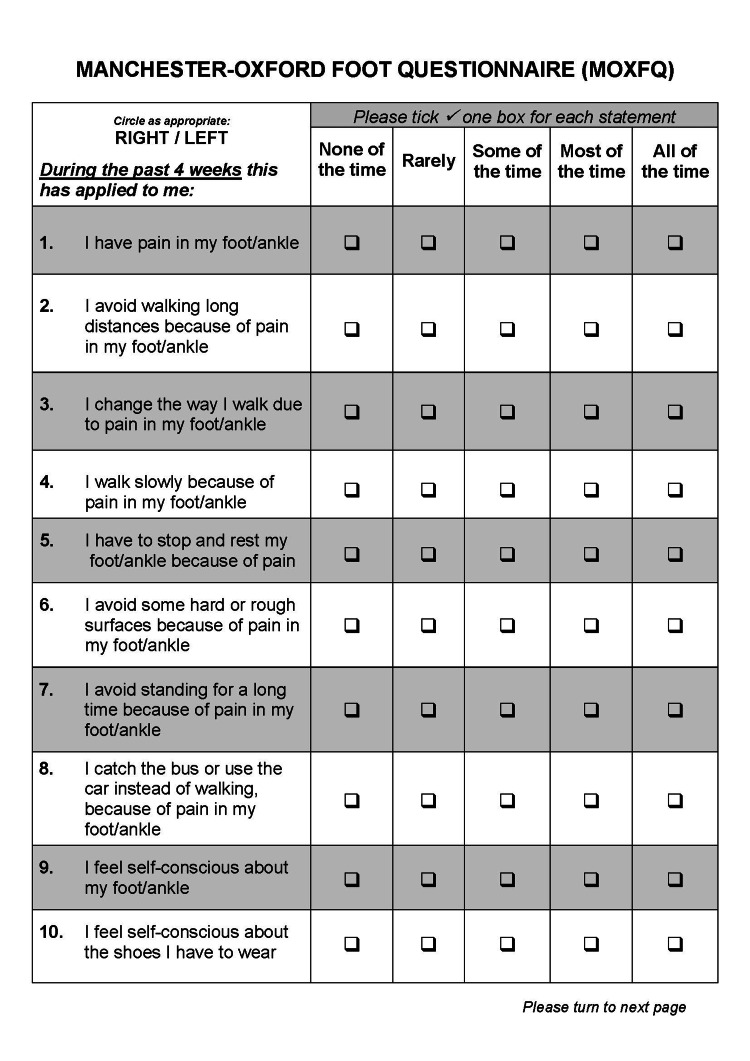
The Manchester-Oxford Foot Questionnaire (MOXFQ) printed survey form for patient reported outcomes: page 1 Figure 5 and Figure 6 were reprinted from [10]. They have been used in this study to calculate the function and pain of the patient's ankle following surgery. The MOXFQ also considers the effects that this has on the patient's day-to-day life.

**Figure 6:**
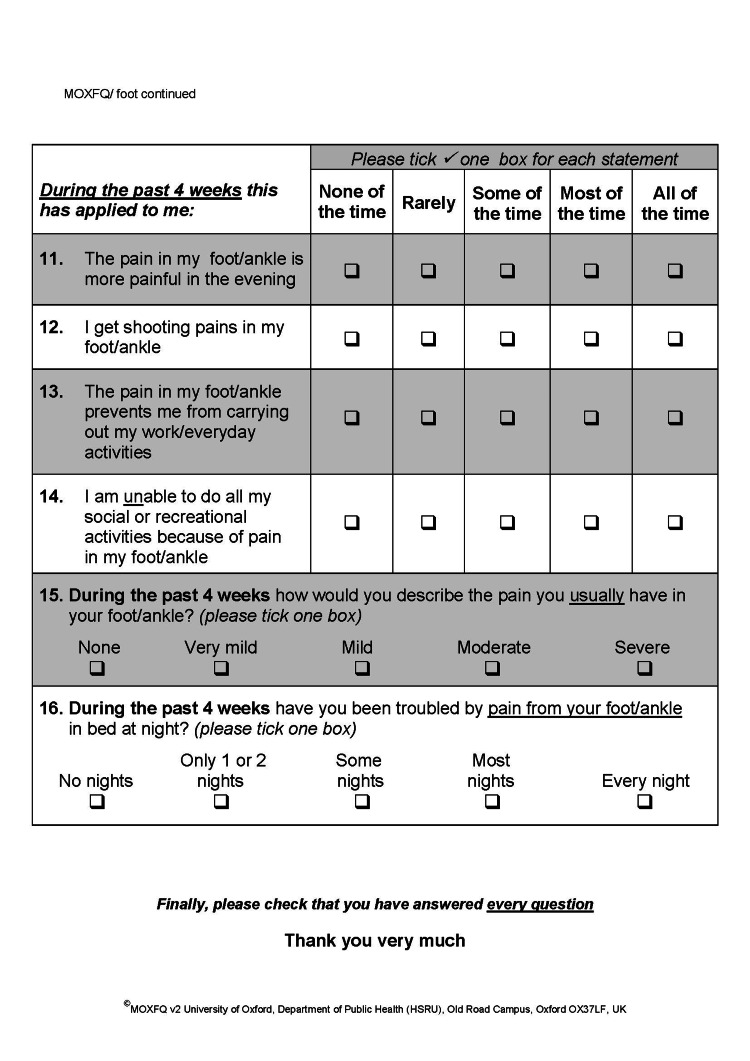
The Manchester-Oxford Foot Questionnaire (MOXFQ) printed survey form for patient reported outcomes: page 2 Figure 5 and Figure 6 were reprinted from [10]. They have been used in this study to calculate the function and pain of the patient's ankle following surgery. The MOXFQ also considers the effects that this has on the patient's day-to-day life.

## Appendices

Appendix A 
